# PicPreview and PicSummary: Two Timesaving Plugins for the Fluorescence Microscopist

**DOI:** 10.3390/cells10040846

**Published:** 2021-04-08

**Authors:** Gabrielle Vieyres

**Affiliations:** 1 Junior Research Group “Cell Biology of RNA Viruses”, Heinrich Pette Institute, Leibniz Institute for Experimental Virology, 20251 Hamburg, Germany; gabrielle.vieyres@leibniz-hpi.de; 2 Integrative Analysis of Pathogen-Induced Compartments, Leibniz ScienceCampus InterACt, 20251 Hamburg, Germany

**Keywords:** Fiji, ImageJ, plugin, macro, microscopy, montage, picture, image, index, fluorescence

## Abstract

This article targets cell biologists who use fluorescence microscopy but lack automatic tools to summarize and manage their image datasets. When using microscopy to document a phenotype, multiple and random pictures are required to reflect the biological diversity of each imaged sample. Managing, integrating and summarizing the acquired data can be a daunting task that becomes extremely time-consuming unless one automatizes it. Unfortunately, if many biologists use microscopy, only a few have automatized procedures to cope with the data generated. For the majority of microscope users, the two developed complementary ImageJ plugins, PicPreview and PicSummary, will allow, in a few clicks and in an instant, to obtain an overview of all pictures taken for each sample of an experiment and a summary with one user-defined representative picture per sample. The plugins and a video tutorial, as well as demonstration pictures, are available as supplementary data at the journal website. PicPreview and PicSummary should save precious time in managing microscopy datasets and in preparing figures for publications.

## 1. Introduction

In the biology field, advanced but also beginner microscopists often face large numbers of images. These are necessary to encompass the heterogeneity of the biological samples, such as complex tissue, primary cell samples or more uniform cell lines. Summarizing, documenting, sorting and communicating the acquired datasets might be challenging tasks. Technological advances in terms of hardware and software have made multiple image acquisition very time efficient. For instance, with a motorized stage, one can automatically acquire and save a series of pictures randomly taken over several samples. Due to the lack of user-friendly and free automatic tools, many users still manually inspect their piles of images and use office programs to summarize their results, which can be overwhelming and time intensive.

ImageJ is an extremely powerful open-source image-processing program initially developed and launched as “NIH Image” in 1987 by a team of scientists from the National Institutes of Health (a list of the contributors for ImageJ and its distribution Fiji is available online at https://imagej.net/Contributors#Fiji, website accessed latest on 6 April 2021) [1]. In particular, ImageJ and its distribution Fiji contain all the necessary tools to present images and summarize them into montages [1,2]. Automatizing these tasks over multiple images or image folders requires scripting skills, a barrier that discourages most microscopists. This article describes two ImageJ plugins whose aim is to make microscopy image management accessible to all in a few clicks. It provides a step-by-step guide, demonstration pictures and a video tutorial for using these tools so that no experience with ImageJ/Fiji is required. The first plugin, “PicPreview,” assembles, for each sample acquired, a montage of all taken pictures, with single channels and merged pictures. The second plugin, “PicSummary,” builds a montage with one representative picture of each condition, as chosen by the user. These plugins function with either default or user-defined settings. The latter allow, for instance, to choose the channels to depict, as well as their colors in the merge, the format of the exported montage or whether the pictures in the montage should be automatically annotated or not. They also incorporate a contrast adjustment tool. The plugins are compatible with a wide variety of image formats originating from all common microscope acquisition software. Tedious tasks that take hours manually can be performed within a few minutes or seconds over hundreds of pictures and with minimal user intervention. These plugins will facilitate the documentation of microscopy results, the choice of truly representative pictures and the building of figures for publications and should save precious time for all those who use microscopy but are not ready to learn how to script. They will also serve as precious templates for users to develop other macros, answering their own needs in terms of image presentation or quantitative image analysis.

## 2. Materials and Methods

### 2.1. Installation of Fiji/ImageJ

**Figure 1:**
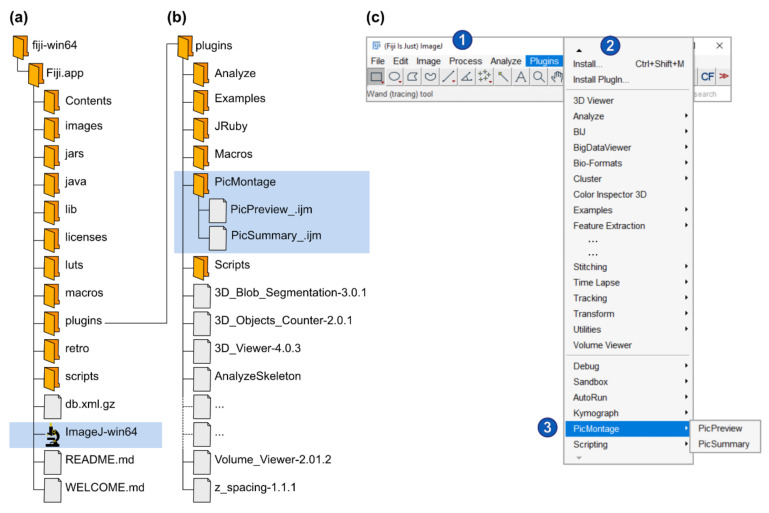

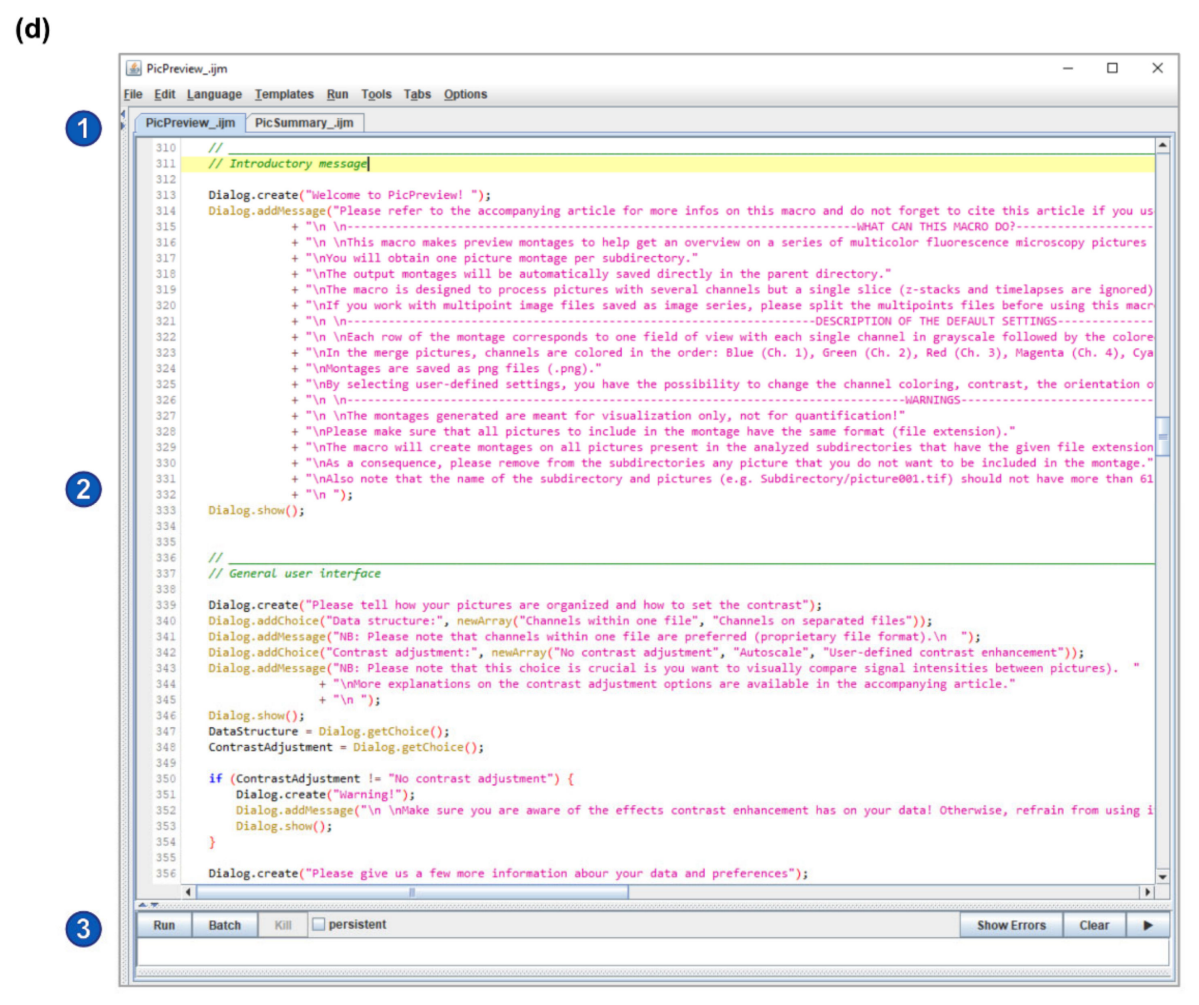
Installation of Fiji and of PicMontage. (**a**) Structure of the Fiji installation files (downloaded from https://imagej.net/Fiji/Download, last accessed on 6 April 2021). The fiji-win64 directory should be copied to the computer hard drive. To start ImageJ/Fiji, simply open the ImageJ-win64.exe file (highlighted in blue). (**b**) To install PicPreview and PicSummary as a plugin package (see Option 1 in the main text), create a folder “PicMontage” (highlighted in blue) in the “plugins” directory, paste PicPreview_.ijm and PicSummary_.ijm there and restart ImageJ/Fiji. (**c**) To run PicPreview and PicSummary as plugins, open the “Plugins” menu (2) from the main window (1) and select PicMontage (PicPreview/PicSummary) (3). Note that this panel reproduces a screenshot of the main ImageJ/Fiji user interface [1,2]. The following figures and panels reproduce screenshots of the different steps of the plugin execution in ImageJ/Fiji. (**d**) Alternatively, to open PicPreview and PicSummary as macros (see Option B in the main text), simply drag the PicPreview_.ijm and PicSummary_.ijm files in the main ImageJ/Fiji window (labeled 1 in Panel (**c**)). The macros will open in the text editor as depicted (2). To shift from one macro to the other, select the appropriate tab (1). Press “run” to execute the selected macro (3).

Fiji is a distribution of ImageJ [1,2] and can be freely downloaded from the internet (https://imagej.net/Fiji/Download, accessed latest on 6 April 2021). Please download and save the zip folder to your computer hard drive and extract all files. Note that you do not need to install the program or have administrator rights to use it. To start Fiji, simply open the extracted folder (fiji-win64\Fiji.app) and double-click on the ImageJ-win64.exe file (Figure 1a). Note that PicPreview and PicSummary were developed with the Windows 64-bit version of Fiji based on ImageJ v1.53c. The author recommends using this version because compatibility issues with more recent versions could arise.

### 2.2. Installation of the PicPreview and PicSummary Plugins

**Figure 2:**
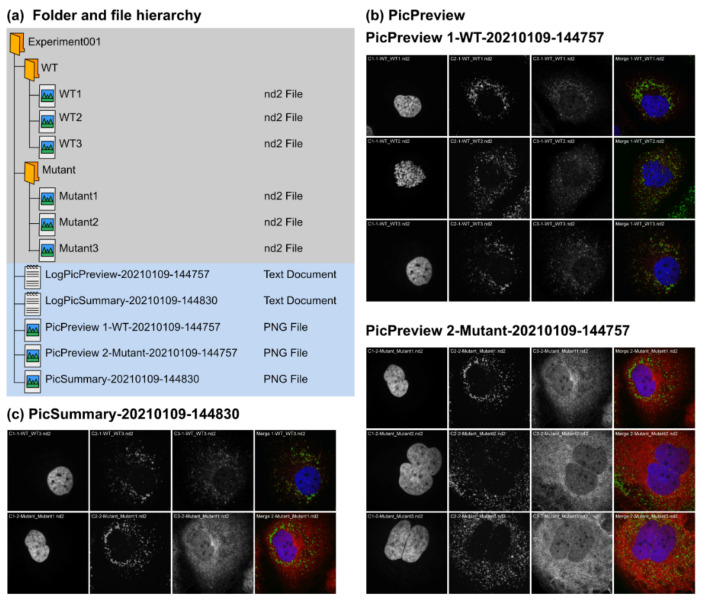

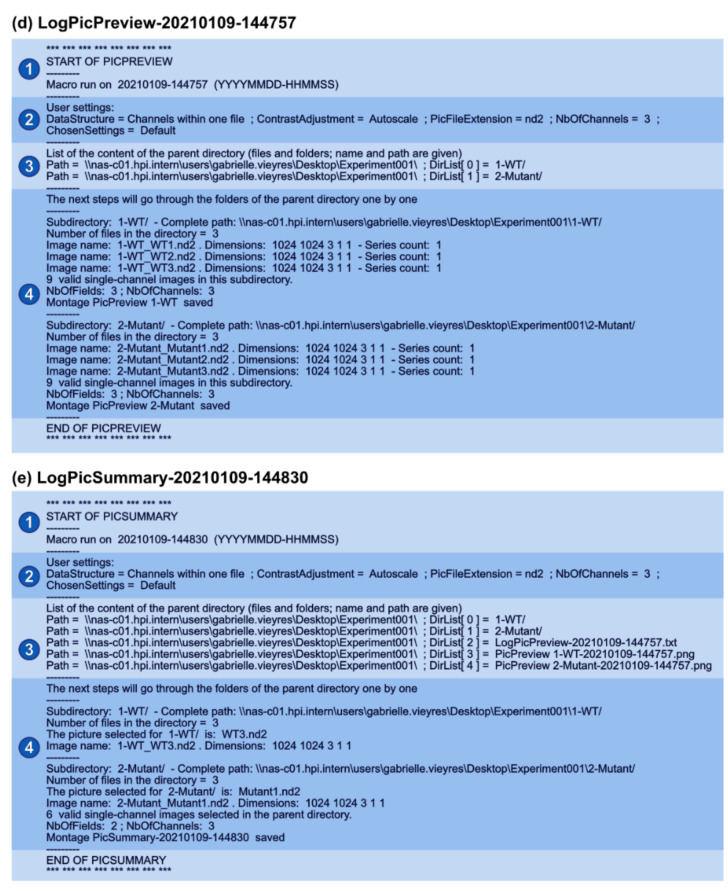
Input and output data for PicPreview and PicSummary. PicPreview and PicSummary were run consecutively and with default parameters on an experimental dataset (Experiment001) containing 2 conditions (1—WT (Wildtype), 2—Mutant) and 3 images per conditions (WT1.tif, WT2.tif, WT3.tif and Mutant1.tif, Mutant2.tif, Mutant3.tif). Note that the plugins and the test dataset are provided as Supplementary Materials (Macro Set “PicMontage” and Dataset “Experiment001”). Image data files are structured with all channels within one file (in this example, each image data file is one .nd2 file with 3 channels). Only the number of channels (“3”), the directory path and the “autoscale” option were edited in the user interface (see Section 3.2). (**a**) Data file structure. Note that the plugins have to be run on the parent folder “Experiment001,” and the pictures should be saved in subfolders within the parent folder (here, “WT” and “Mutant”). The output files (log files and montages) are saved directly in the parent folder (“Experiment001”). Output files are tagged with a timestamp (YYYYMMDD-HHMMSS) so that log and montage files can be unequivocally paired. (**b**,**c**) Montage outputs with PicPreview and PicSummary using standard settings. In both cases, note that the images in the montages are labeled with the channel number, the folder and the file name, in this order. Please also note that the font size of the image labels might vary depending on the picture pixel dimensions. With the advanced settings (see Section 3.3), it is possible to omit these labels. (**c**) Montage output with PicSummary using standard settings. WT3.nd2 and Mutant1.nd2 were chosen as representative pictures for Wildtype and Mutant, respectively. (**d**,**e**) Log files for PicPreview (**d**) and PicSummary (**e**). The structure of the log files is similar for both tools. An introductory message (1) includes the macro used and the execution date (timestamp as above). Section (2) records the user inputs and chosen parameters. This is important to record for instance any contrast adjustment that might have been applied to the pictures for the montages. Section (3) lists the content of the parent directory (in this case, Experiment001). Finally, Section (4) lists for each subdirectory the selected image files used for the montage (all images following the user criteria for PicPreview, representative images for PicSummary).

PicPreview and PicSummary are available as supplementary materials to this article (Macro Set “PicMontage”). A small dataset to test the plugins is also provided (Dataset “Experiment001”) and was used to create Figure 2. PicPreview and PicSummary were written as macros in the ImageJ Macro Language (IJM), a scripting language built into ImageJ. There are two easy options to use PicPreview and PicSummary. They can be run either as macros or as plugins. The first option (Option A) is optimal for the pure usage of the macros; the script is not visible, which avoids inadvertent edits by the user. In addition, Option B allows the user to edit, personalize and eventually extend the macro or troubleshoot any potential issues.

Option A: Open the Fiji program “plugins” folder (Fiji.app\plugins). Create a new directory called “PicMontage” and paste the macro files PicPreview_.ijm and PicSummary_.ijm into this directory (Figure 1b). Restart Fiji. In the Fiji menu bar, select “Plugins” and scroll down until you find “PicMontage” (it might be at the bottom of a very long list) (Figure 1c). Within “PicMontage,” select PicPreview or PicSummary, and the macro will start. The macros will remain available in the “Plugins” menu every time you restart Fiji.

Option B: Save the macro files PicPreview_.ijm and PicSummary_.ijm on your computer. Drag and drop the files in the Fiji main window. The macro will open in a text editor (Figure 1d). Click on the “Run” button to execute the macro. Note that you will need to reopen the macro every time you start Fiji and want to use the macros.

## 3. Results

### 3.1. Overview of PicPreview and PicSummary

PicPreview and PicSummary are two complementary tools based on the same architecture (Figure 2, Supplementary Data Macro Set “PicMontage”). From a directory (experiment) containing several subdirectories (tested conditions/coverslips/samples) with pictures, they deliver picture montages and associated log files (Supplementary Dataset “Experiment001” and Figure 2a). Typically, the user will first use PicPreview to get an overview of all taken pictures in an experiment. PicPreview provides one summary file per condition tested (for each condition, it generates one montage, gathering all images taken; Figure 2b). This way, it is possible to compare, with one look, individual channels or their overlap across the different fields of view. This can facilitate comparing conditions, drawing conclusions and selecting representative images for each tested condition. In a second step, PicSummary produces a digest of the experiment (Figure 2c), with one user-defined picture per condition (representative picture). This summary is useful to communicate results in a condensed fashion (for lab book records or in oral communications) and can serve as the basis to create figures for publications. The log files are useful to document the montages’ parameters and for eventual troubleshooting if the plugins would not deliver the expected results (Figure 2d,e). Note that all output files (montages and log file, see the files highlighted in blue in Figure 2a) are saved in the directory but will not directly appear in ImageJ/Fiji when running the plugins, they need to be actively opened by the user for consultation. A short tutorial on the use of PicPreview and PicMontage is provided in Video S1.

### 3.2. User Interface, Minimal User Input and Default Settings

**Figure 3:**
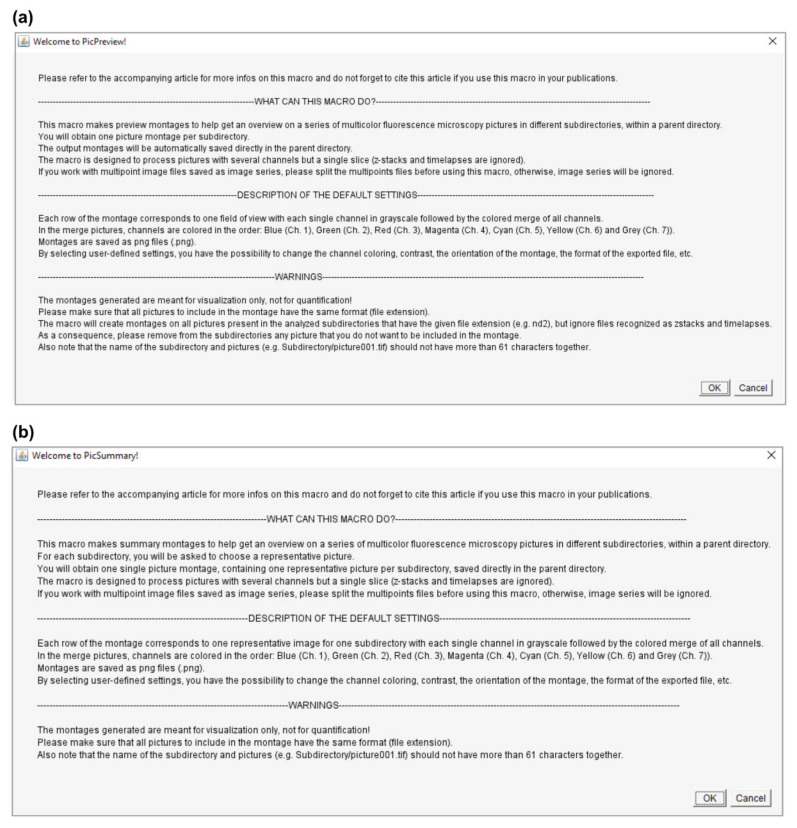
Introductory messages for PicPreview (**a**) and PicSummary (**b**).

**Figure 4:**
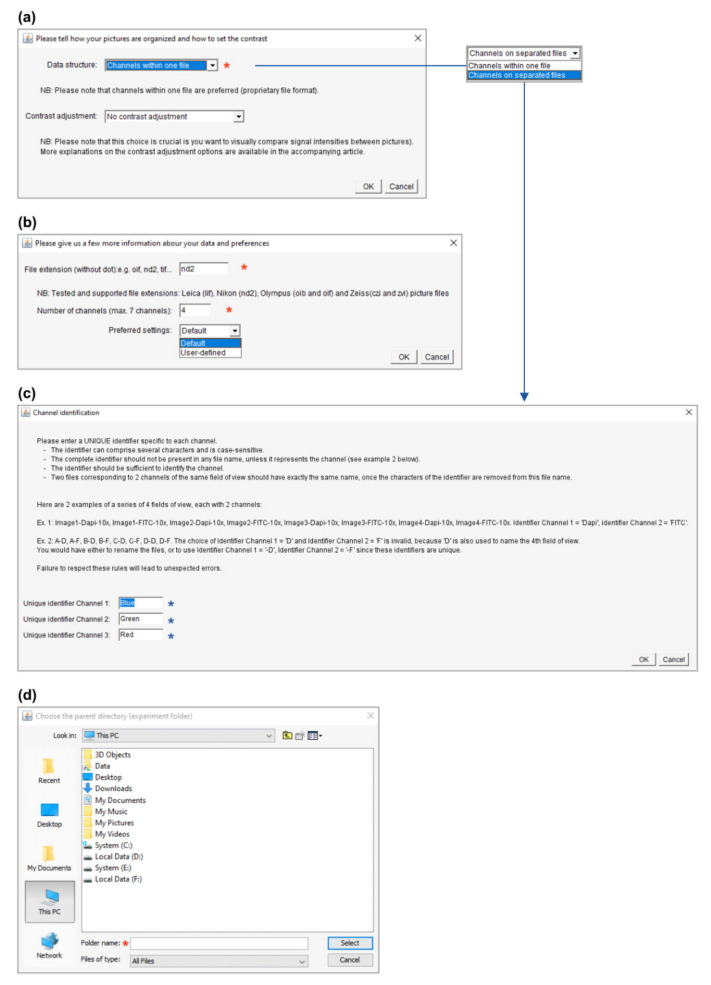
Basic user interface and user input collection. The panels are depicted in their order of appearance when running PicPreview or PicSummary. (**a**) As a first step, please describe whether the channels of your multicolor images are saved together (data structure = “Channels within one file”) or separately (data structure = “Channels on separated files”). In this window, you also have the possibility to choose between several contrast enhancement options (see Section 3.3.1). (**b**) Now indicate the file format and the number of channels in your files (any file not complying with these criteria will be ignored from the montages). At this stage, choosing “User-defined” settings will open additional windows and personalization options as described in Figure 5c–e and Section 3.3.2. (**c**) In case the dataset is structured with “Channels on separated files,” an identifier must be given to distinguish each channel, obeying the described rules. (**d**) Finally, please select the dataset’s location and remember to select the parent directory and not the directory directly containing the pictures (see Figure 2a, where “Experiment001” was selected).

Both macros use a similar user interface and require minimal input from the user. After an introductory message (Figure 3), several windows will open consecutively to ask for basic information on the image dataset. This consists basically of four pieces of information (noted with a red asterisk in Figure 4): (i) the structure (Figure 4a) and (ii) the extension (format) of the image data files (Figure 4b), (iii) the number of acquired channels (e.g., “3” for DAPI/GFP/RFP channels; Figure 4b) and, finally, (iv) the location (path) of the folder containing the experimental data (Figure 4d). The structure of the image data files depends on the saving mode chosen during image acquisition. With most modern microscope software, it is possible to save multichannel images as a single file (data structure = “Channels within one file”), but some more basic programs do not offer this option, and the user saves each channel independently (data structure = “Channels on separated files”). In this last case, the user is also required to enter a meaningful identifier for each channel (see the blue asterisks in Figure 4c). Please consider the identifier requirements (see explanations in Figure 4c) when naming your images. In addition to this basic information, the user interface also proposes multiple options to personalize the output, as described in Section 3.3.

ImageJ/Fiji includes Bio-Formats [3], a software tool from the Open Microscopy Environment (OME) consortium [4] able to read over 150 file formats (https://www.openmicroscopy.org/bio-formats/, accessed latest on 6 April 2021). Note that the plugins were tested with the main commercial microscopy acquisition software and with typical image formats, as depicted in Table 1. If for any reason the image files were not successfully opened, try and export them in another format. Please also note that any image file having a different extension or number of channels than defined by the user, and any image file having more than one slice (z-stack), frame (time-lapse), or point (multipoint file or image series) will be ignored. If you would like to process such image files, individual slices, frames or points first need to be extracted.

### 3.3. Advanced Settings

#### 3.3.1. Contrast Enhancement

**Figure 5:**
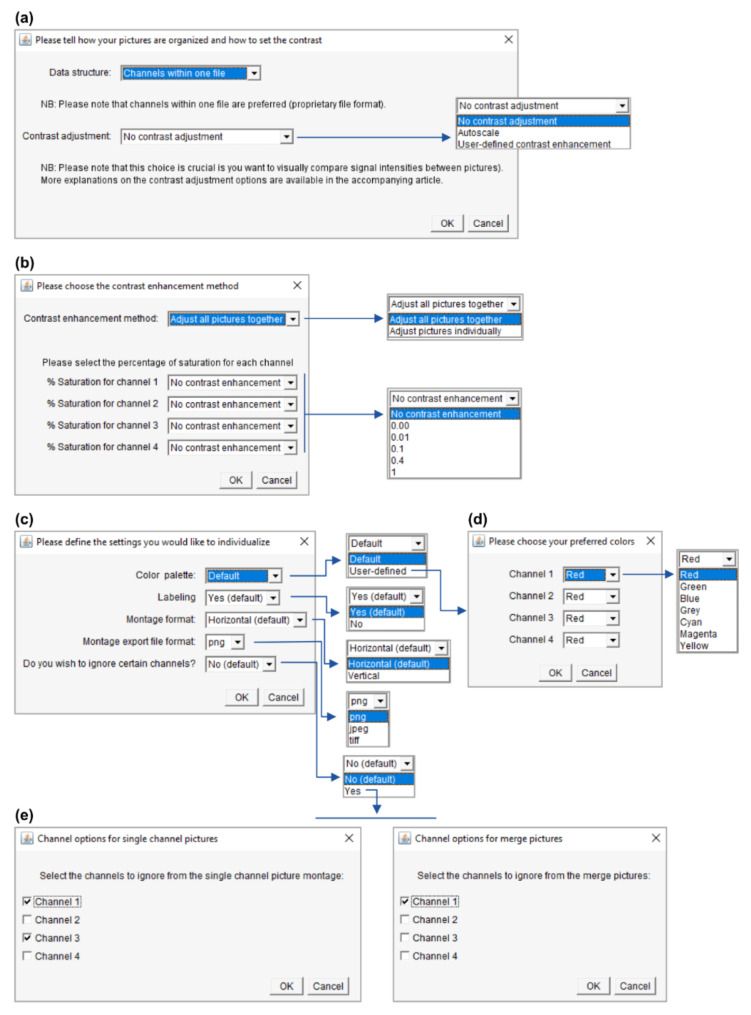
Advanced settings. The panels are depicted in their order of appearance when running PicPreview or PicSummary and illustrate a dataset with 4-channel pictures. (**a**) Choosing the “user-defined contrast enhancement” method will lead to the window in Panel (b) opening. (**b**) Further contrast enhancement options (please see the main text, Section 3.3.1). (**c**) Options to individualize the montages (this window appears when the user selects “user-defined” preferred settings in the window depicted in Figure 4b). If you select the user-defined color palette, the window in Panel (**d**) will open. If you choose to ignore channels, the windows in panel (**e**) will open. (**d**) Selection of color for each channel. (**e**) Here you could choose to omit certain channels from the single-channel pictures or the merge pictures.

**Figure 6:**
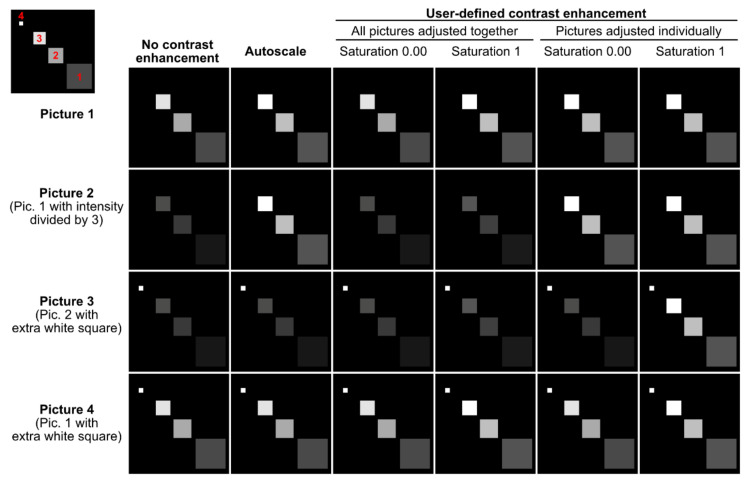
Contrast enhancement pitfalls. This figure represents 4 artificial pictures (rows) after applying different contrast enhancement methods (columns). Each picture contains 3 to 4 squares with different signal intensities (see the top left corner where these squares are numbered in red). The first column represents the raw images. The largest square 1, for instance, has the same mean signal intensity in Pictures 1 and 4 but a 3-fold lower mean intensity in Pictures 2 and 3. In Picture 3, the little square 4 is brighter than any other square. The figure shows how different contrast enhancement methods can make such inter- and intrapicture intensity comparisons fully invalid.

A set of options are available to personalize the montages, as described in Figure 5. First of all, it is possible to adjust the image contrast (Figure 5a). This can be very useful for visualization purposes if some channels do not use the whole dynamic range of the image and some pictures or structures are very dim. Contrast enhancement is based on histogram stretching. For a 16-bit 1024 × 1024 pixel image, each of the 1,048,576 pixels has an intensity value between 0 (black pixel) and 65,536 (2^16^, white pixel). This can be represented as a histogram depicting the number of pixels (*Y*-axis) for each gray value (*X*-axis), which can be easily visualized in ImageJ/Fiji (Analyze/Histogram). In practice, the pixels might not distribute on the whole histogram, resulting in low contrast. Contrast enhancement stretches the image’s pixel intensity range to use the whole span of available pixel intensities (e.g., 0–65,536 for a 16-bit image or 0–256 for an 8-bit image). However, contrast enhancement should be applied with care as it often precludes subsequent signal intensity comparisons (between pictures or even between structures within a picture, if some structures have a saturated signal). Figure 6 highlights some of these pitfalls.

With the “autoscale” option (Figure 5a), the intensity range of each picture and of each of its channels is stretched to use the whole bit depth of the image. With this method, no intensity comparison can be made between images, and images with only a background signal will be artificially enhanced until the brightest background pixels are white. The “user-defined contrast enhancement” method (Figure 5a) allows more freedom (Figure 5b). Channels are treated separately, and one can choose to adjust the contrast of all pictures (belonging to a montage file and a channel) together or individually. In the first case, the highest intensity pixel among all treated pictures will serve as a reference. On the opposite, when choosing to adjust the pictures individually, each picture’s highest intensity pixel serves as a reference (and again, a background will show as a signal). Furthermore, the percentage of saturation can be set for each channel to 0, 0.01, 0.1, 0.4 or 1% and individual channels can be kept without contrast enhancement.

Let’s imagine an experiment to test the percentage of GFP-positive cells in different conditions (treatments). DAPI serves as a counterstain but the different treatments are not expected to affect the DAPI signal. If the DAPI staining is dim, it is possible to select “user-defined contrast enhancement” and choose a saturation value for Channel 1 (DAPI). With a saturation value of 0.00, Fiji will enhance the contrast but avoid saturation (provided that the raw images have no saturated pixels). However, there might be a few outlying bright pixels in the image caused by imaging or staining artifacts or by real biological structures which are not the structures of interest. For example, a minority of cells might undergo mitosis and show saturated DNA signals. Allowing a certain percentage of saturation (0.01, 0.1, 0,4 or 1%) is a useful possibility to increase the contrast of the majority of cells for visualization purposes if they are the cells of interest. For the DAPI channel, it might not be important whether one adjusts “all pictures together” or the “pictures individually,” as it only serves to localize the cells. For the GFP channel (Channel 2); however, it is crucial to “adjust all pictures together”; otherwise, no comparison can be made between the pictures. The user can choose to keep the raw images (“no contrast enhancement”) or enhance their contrast with an appropriate saturation value.

#### 3.3.2. Other Options

It is also possible to personalize the montages. This can be particularly useful to prepare figures for publications or presentations. For instance, the user can create their own color palette using seven available colors (red, green, blue, gray, cyan, magenta and yellow) and choose to label the montage (image and directory names will appear, which can be useful for lab book records) or not (to have clean montages to be labeled in a more flexible illustration software, for example for figure preparation). The user can also choose between horizontal montages, with the rows corresponding to the images and the columns to the channels/merge, or vertical montages. Choosing png (Portable Graphics Format) and in particular jpeg (Joint Photographic Experts Group) export file formats will fasten the montage saving process (but jpeg compression is lossy and will deteriorate the image quality). Finally, the user can choose to eliminate specific channels from the montage, either by not showing them as single-channel grayscale pictures or by not incorporating them in the merged pictures. This might be useful, for instance, to have a smaller montage by showing the nuclear stain channel only in the merge/only as a grayscale picture if its purpose is simply to localize the cells.

### 3.4. Execution Time

Execution time of the plugins will depend largely on the hardware and on the file format used to export the montages. For instance, the jpeg format will result in montages file sizes around 10 times smaller than the png format, thereby shortening proportionally the file saving time. It is also important to save the images to process directly on the computer hard drive rather than on a remote server. Note that the execution time is registered at the end of the log file (e.g., “Macro finished on 20210311-102933 (YYYYMMDD-HHMMSS) and lasted 34.761 s”). 

The user can appreciate the plugin execution time by looking at several examples in the tutorial video (Video S1). A further example of execution times for two larger datasets is given in Table 2. Using PicPreview and exporting the montages as jpeg files, it was possible to process 30 folders each with 10 four-channel pictures in 3–4 min (Dataset 1, total of 1500 images compiled into 10 montages) and 2 folders each containing 20 four-channel pictures in 35 s (Dataset 2, total of 200 images compiled into 2 montages). For each dataset, the user working time (time actually requiring user intervention) did not exceed 10–30 s if independent of the number of images processed. As a comparison, the author needed 1 min and 48 s of working time to transform a single four-channel picture into a montage (a total of five images compiled into one montage), and this time has to be multiplied by the number of images processed.

Overall, as measured on these two large datasets (Table 2), PicPreview executed in under 1.6 s per treated single-channel/merge image when saving the montages as png files and under 0.2 s per image when saving the montages as jpeg files. Most of the processing time was spent by the computer to save the montages—it would also be needed if the user was to process the images manually. The time to “click through” the plugin might vary from 10 to 30 s when using the default settings; it can be longer if the user extensively changes the parameters, but it is unlikely to exceed 1 min per execution. Furthermore, the plugins only need user intervention at the beginning of their execution. This user working time is included in the depicted execution time. Note that the complete execution time is much shorter for PicSummary than PicPreview because PicSummary only takes one image per subfolder. Here, the plugin execution time depends more on the user’s speed in selecting the representative images for each sub-folder. For these examples (Table 2), the first image was always selected as a representative image for each folder, minimizing this selection time.

In summary, although these time measurements will vary depending on the user hardware, PicPreview and PicSummary only take a minute fraction of the time required for manual processing of image data (see also the examples in the supplementary tutorial, Video S1). Using the plugins for these tasks is not only a huge time saver but also has the advantages of preventing user mistakes and automatically documenting the used parameters in a log file.

### 3.5. Troubleshooting

#### 3.5.1. Interruption of the Macro/Plugin with an Error Message

Given the multiplicity of image formats and different operating systems, it can unfortunately not be ensured that the plugins will run without any mistake in all tested cases. First of all, please test the plugin on the available test folder to ensure there is no incompatibility with your system. Secondly, make sure that your dataset complies with the criteria described in the plugin user interface and in this publication. Unfortunately, in case of an interrupted plugin, no log file will be saved. However, a “Macro Error” window might appear. The experienced user can track down the failing command by running the .ijm file from the Fiji text editor (as described in Figure 1d), activating the “Show Debug Window” function in the “Macro Error” window and inspecting the script to troubleshoot it. It might also be helpful to remove or inactivate the line “setBatchMode(true);” in the script (with this line active, the images remain hidden during the script execution, making it much faster) so that images become visible during the macro execution, making it easier to pinpoint the step leading to the error. Verify that the images are properly opened. If it is not the case, there might be an issue with the image data files, and it might help to export the data files as tif files, making sure each file is a simple stack with only multiple channels (no time series or z-stack). Make sure that the picture name is not too long: in the author’s experience, the subdirectory and picture names together (Subdirectory/picture001.ext, where ext is the image file extension) should have no more than 61 characters, otherwise it can cause bugs, but this could also depend on the operating system.

#### 3.5.2. Unexpected Montage Output or no Montage Output

It is important to critically inspect the obtained montages so as to detect any unexpected behavior. Unexpected results might be tracked back to the plugins themselves or to any built-in function used by the plugins. They might also depend on the image data files and due to the countless possibilities for input/plugin parameters constellations, it, unfortunately, cannot be guaranteed that the plugins are completely bug-free. With some image formats, ImageJ/Fiji bit depth autoscaling might alter the display of the opened image, an issue that can be circumvented by changing some settings in Fiji. If the user is used to particular settings to visualize their data files, they should make sure that these settings are not affected by the plugins.

If the plugins’ output is not as expected (or there is no montage file as the output), consulting the log file might help troubleshooting. It might also be useful to rerun the plugin using the default settings. One frequent issue when using the macro on pictures with separated channels is the improper naming of the images (the channel identifier should be unique and the only set of characters that differs between files of several channels of the same picture, please see Figure 4c). If the image files are not properly opened or processed, it might help to export them in a different format (e.g., tif files), making sure that they have only three dimensions (*x*, *y* and channels). Commercial microscope software usually include a batch export function to export all acquired pictures as tif in one go and split image files according to one particular dimension (e.g., split z-stacks or multipoint files/image series).

## 4. Conclusions

This article presents a toolset that will allow microscopists to process their multicolor fluorescence images quickly and easily. It aims at facilitating image analysis for daily fluorescence microscopy usage. Time previously lost in manually merging multicolor images and assembling image panels can be better invested in experimental design, image acquisition and quantification. For experienced users, it is a solid basis for the development and extension of their own scripts because the complete source code is available. For instance, the macros can serve as a starting point for the user interested in iterative image analysis over multiple data files and folders. This way, it might quicken the learning process of self-learners who want to make the best of the possibilities offered by Fiji/ImageJ [1,2].

## Figures and Tables

**Table 1:** Image data files tested for compatibility with PicPreview and PicSummary.

Microscope Company	File Format
Nikon	nd2
Olympus	oif
Zeiss	czi, zvi
Standard image files	tif

**Table 2:** Indicative recorded execution times for PicPreview and PicSummary on two large datasets. Note that these times will depend on the computer system used (in this case, a laptop equipped with Intel^®^ Core™ i7-10750H CPU, 16 GB RAM, 512 GB SSD).

Dataset	Plugin—Montage Output	Export File Format	Execution Time
Dataset 1: 30 subfolders each containing 10 four-channel pictures (300 multichannel images)	PicPreview—30 montages each containing 50 images (10 fields of view with 4 single-channel pictures and one merge picture)	png	37 min (1.48 s/pic)
		jpeg	3.4 min (0.14 s/pic)
	PicSummary—1 montage containing 150 images (30 fields of view with 4 single-channel pictures and one merge picture)	png	4.5 min
		jpeg	74 s
Dataset 2: 2 subfolders each containing 20 four-channel pictures (40 multichannel images)	PicPreview—2 montages each containing 100 images (20 fields of view with 4 single-channel pictures and one merge picture)	png	5.3 min (1.59 s/pic)
		jpeg	35 s (0.18 s/pic)
	PicSummary—1 montage containing 10 images (2 fields of view with 4 single-channel pictures and one merge picture)	png	30 s
		jpeg	17 s

## Data Availability

All relevant data is contained within the article or supplementary material.

## Supplementary Materials

The following are available online at https://www.mdpi.com/article/10.3390/cells10040846/s1, Dataset “Experiment001,” Macro set “PicMontage,” Video S1: Tutorial for the use of PicPreview and PicSummary.
