# Supplementary figures and images for: PicPreview and PicSummary: Two Timesaving Plugins for the Fluorescence Microscopist
Source: Cells. 2021 Apr 8;10(4):846. doi: 10.3390/cells10040846 (PMC8068365; doi:10.3390/cells10040846)

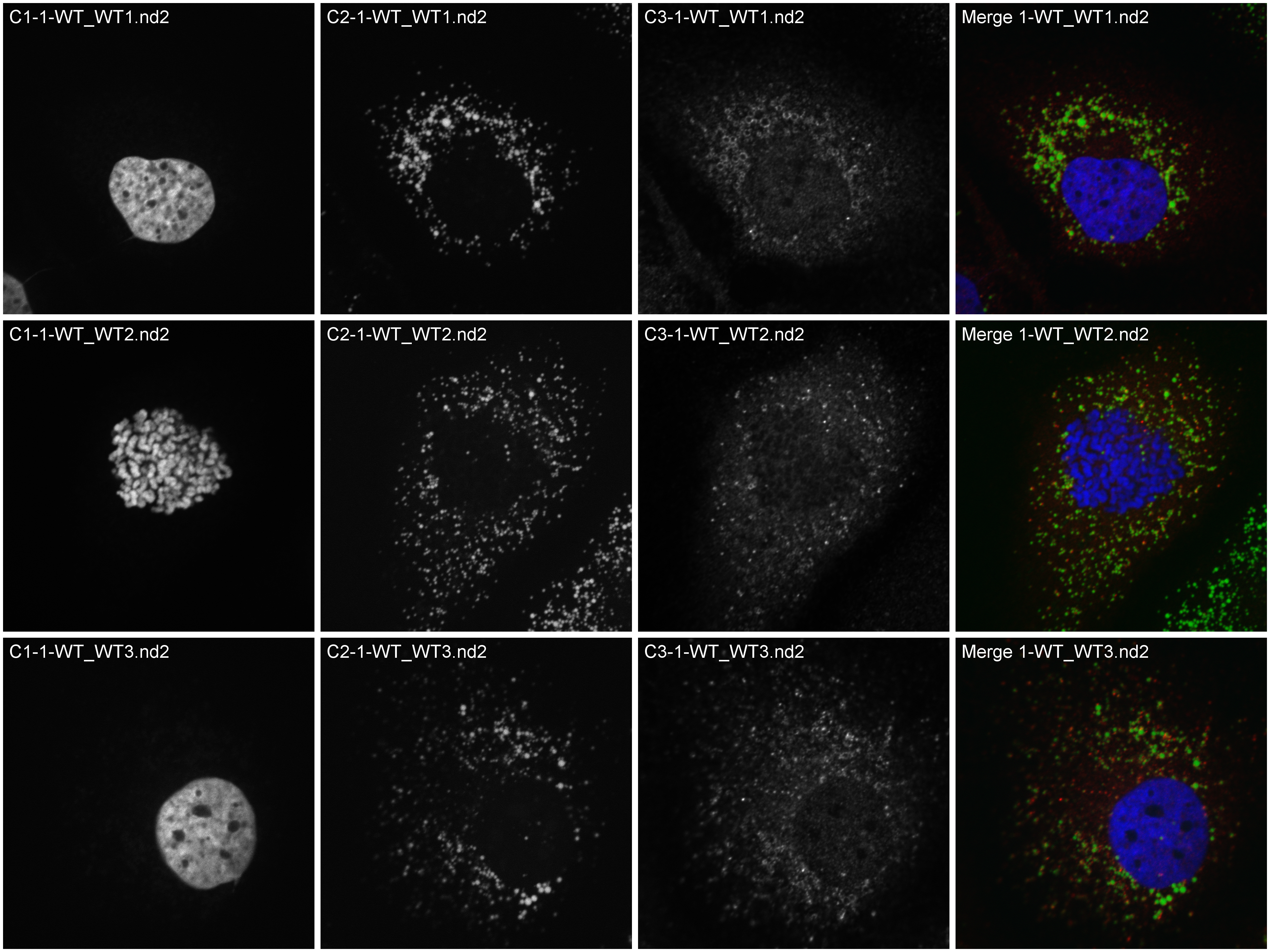

Supplement: Supplementary file 1 [file cells-10-00846-s001.zip › Supplements/Experiment001/PicPreview 1-WT-20210109-144757.png]

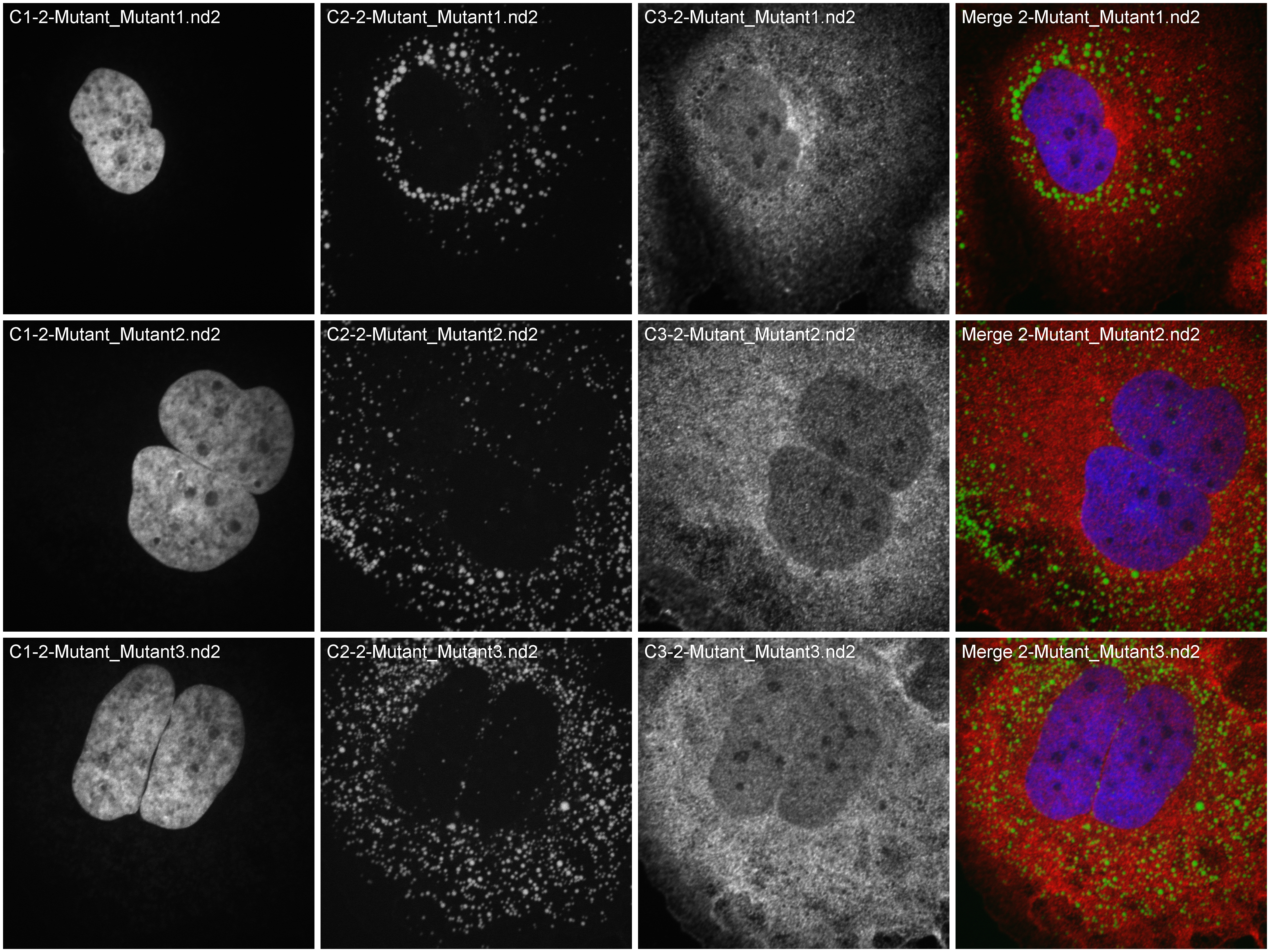

Supplement: Supplementary file 1 [file cells-10-00846-s001.zip › Supplements/Experiment001/PicPreview 2-Mutant-20210109-144757.png]

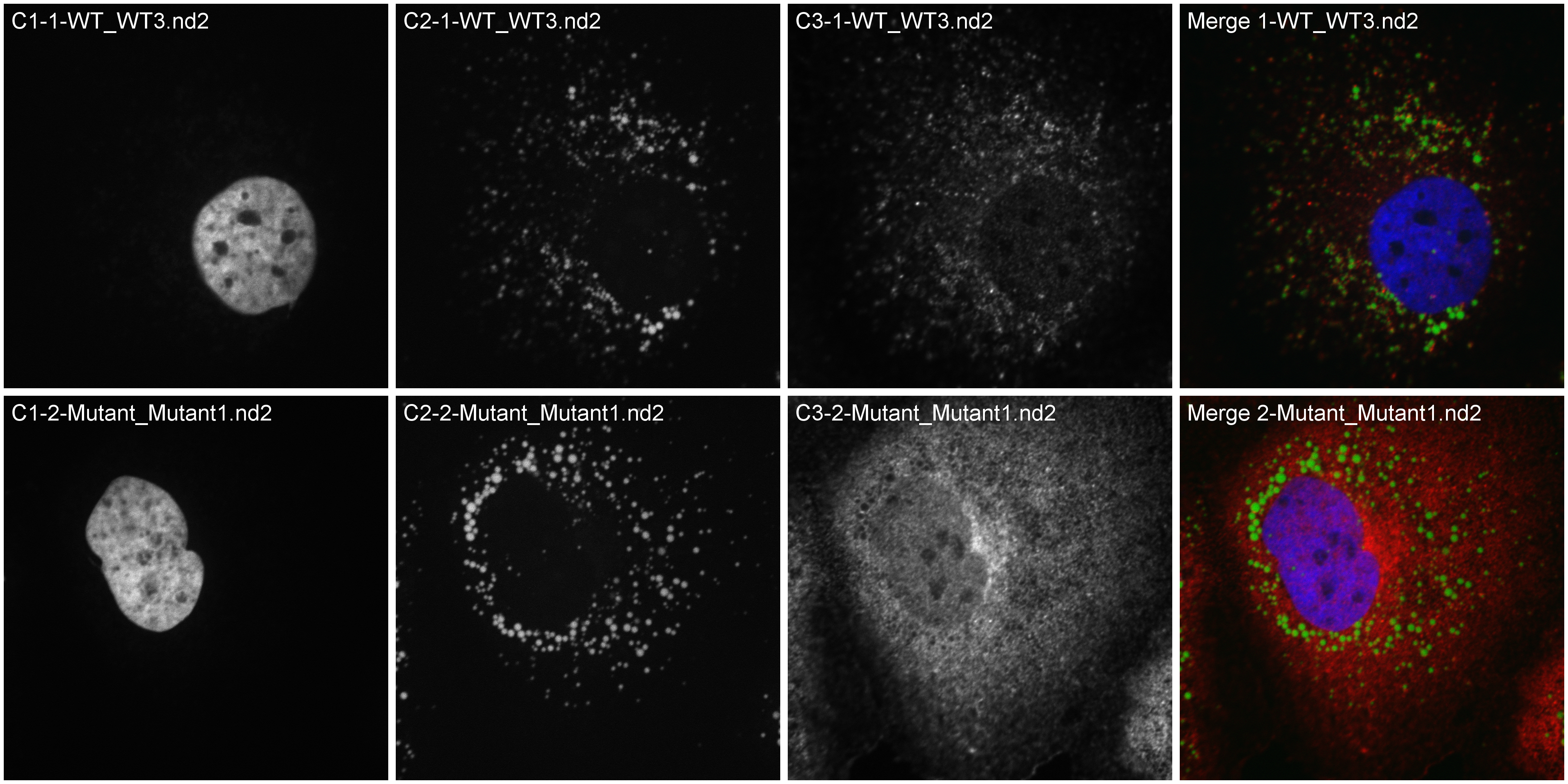

Supplement: Supplementary file 1 [file cells-10-00846-s001.zip › Supplements/Experiment001/PicSummary-20210109-144830.png]
